# End of life care preferences in the Arab population in Israel– bridging the gap between unfounded assumptions and autonomous wishes

**DOI:** 10.1186/s12910-025-01201-9

**Published:** 2025-04-04

**Authors:** Morad Sayid Ahmad, Maya Peled Raz

**Affiliations:** University of Haifa, Haifa, Israel

**Keywords:** End-of-Life care, Arab community in Israel, Muslim medical ethics, Advance care planning, Cultural barriers

## Abstract

**Introduction:**

End-of-life (EOL) decision-making involves complex ethical, cultural, and religious considerations, particularly within minority communities. In Israel, the Arab population, comprising approximately 21% of the country’s population, remains underrepresented in EOL research. This study explores the EOL care preferences of elderly Arab individuals and their families, focusing on the interplay between cultural values, religious beliefs, and personal autonomy.

**Methods:**

A qualitative study was conducted using semi-structured interviews with 24 participants, including elderly individuals (aged 60+) and their family members. Participants were recruited through purposive and snowball sampling in community settings across northern Israel. Data were transcribed, translated, and thematically analyzed to identify key patterns in attitudes toward EOL care.

**Results:**

Findings reveal a strong preference among elderly Arab participants for a peaceful and dignified death at home rather than in a medicalized setting. Quality of life was prioritized over life-prolonging treatments, with religious beliefs playing a significant role in shaping perspectives. However, cultural taboos and generational differences hindered open communication within families. Many younger family members assumed their elders preferred life extension, while elderly participants often desired comfort-focused care. Additionally, a lack of awareness of advance care planning tools limited the ability of patients to formally express their preferences.

**Discussion:**

A major challenge identified in this study is the absence of open discussions about EOL preferences, driven by cultural taboos, emotional discomfort, and fear. Many participants avoided such conversations due to beliefs that discussing death invites misfortune or imposes an emotional burden on loved ones. Younger family members, in particular, hesitated to engage in these discussions, leading to decisions based on assumptions rather than explicit patient wishes. Encouraging structured, culturally sensitive conversations and increasing awareness of advance care planning could help ensure that patients’ preferences are recognized and respected.

**Conclusions:**

Bridging the gap between assumptions and actual preferences requires culturally sensitive communication, increased awareness of advance care planning, and structured family discussions. These measures will ensure that EOL care respects both individual autonomy and cultural values, fostering a more inclusive and patient-centered healthcare approach.

**Supplementary Information:**

The online version contains supplementary material available at 10.1186/s12910-025-01201-9.

## Introduction

End-of-life (EOL) decision-making is increasingly viewed as a core issue within bioethics, raising questions about autonomy, ethical responsibility, and the implications of cultural values on patient care [1]. EOL care decisions impact patients, families, and healthcare providers, intertwining medical and ethical considerations. Patient preferences for EOL care have been widely studied, with increasing focus on their intersection with cultural and religious beliefs. Within Israel’s Arab population, these issues are particularly poignant due to the rich cultural and religious tapestry that influences daily life and shapes individual views on death and dying.

Israel’s EOL care is governed by three main laws: the Patient Rights Act (1996), the Dying Patient Act (2005), and the 2016 amendment to the Legal Capacity and Guardianship Law, establishing a framework for advance directives and powers of attorney [2–4]. These laws are intended to safeguard patients’ rights to choose their own treatment paths, thus reflecting a shift from paternalism toward a more autonomy-centered approach. However, it has been argued that such legislative frameworks do not sufficiently address the nuanced needs of minority populations in Israel, including Israel’s Arab community [5, 6].

In 2023, the Arab community comprised roughly 21% of Israel’s population, with 50% living in the northern region and 86% identifying as Muslim [7]. Over 50% of the Arab community in Israel described themselves as “very religious” or “religious” (in contrast to around 21% among the Jewish population) [8]. This is a unique population, constituting a large traditional minority within a largely secular western country. It has gone through ever increasing cultural transitions, impacting many life facets [9].

Research consistently shows that religiosity is a key predictor of attitudes toward forgoing life extension. Generally, the more religious a person is, the more firmly they oppose the cessation or withholding of life-prolonging measures [10]. Consequently, traditional and more religious ethnic groups avoid issuing advance directives, adhering to the belief that the timing and manner of death rest solely in the hands of God [10, 11].

Among Muslims, EOL care involves a delicate balance between the sanctity of life and acceptance of death. In the Islamic tradition, the sanctity of life is a foundational principle, with illness viewed as natural suffering that atones for sins, rewarding both patient and family. Saving a life and providing care are considered among the highest duties. While Muslims believe that God is the ultimate healer of all ailments, they are also obligated to seek treatment whenever possible and must not end life prematurely [12]. While Islamic teachings affirm that life is a gift from God, and as such, it should be respected and preserved, they also acknowledge that suffering should not be prolonged needlessly when death is imminent. Islamic teachings view death as a transition rather than an end [13], leading some adherents to accept that prolonging life at all costs may not align with divine intent, particularly when suffering is involved [14, 15].

While research and literature on EOL decision-making among the Arab population in Arab countries have expanded in recent years, the Arab community in Israel, predominantly Muslim, remains underrepresented in EOL studies. Consequently, little is known about their treatment preferences and decision-making processes in the final stages of life.

The aim of this study is to examine EOL care preferences among elderly Arabs in Israel and their family members. Specifically, it seeks to explore how cultural, religious, and familial factors shape EOL decision-making and care.

## Methodology

### Study design

Informed by a phenomenological epistemological framework, this study seeks to understand participants’ lived experiences and the subjective meanings they attribute to EOL preferences. This framework is particularly suited to exploring complex and culturally embedded phenomena, such as familial dynamics and religious influences on EOL decision-making [16].

### Participants and sampling

Participants included elderly Arab individuals aged 60 and above, living in the community, and their family members who were actively involved in their care. Recruitment utilized a combination of purposive sampling [17]and snowball sampling [18]. To recruit participants for the adult cohort, we collaborated with social workers at elderly community centers located in major Arab or mixed cities in northern Israel. The social workers were tasked with introducing the study to relevant potential participants deemed competent to provide informed consent. If individuals expressed preliminary interest in learning more about the study, the social workers obtained and shared their contact information with the research team for follow-up.

In subsequent communication, potential participants were provided with a comprehensive information sheet and a verbal explanation of the study’s objectives and procedures, confidentiality measures, and their right to withdraw at any time. Those who consented to participate were scheduled for an interview. Following the interview, participants were asked to identify a family member they would designate as their representative for healthcare decisions, should they become unable to make such decisions independently. With the participant’s consent, the designated family member was contacted by the principal investigator, who provided a detailed explanation of the study—both in writing and orally—and invited them to participate.

Furthermore, all participants, whether from the adult cohort or their family members, were encouraged to recommend additional participants who met the study’s criteria. This approach enabled the research team to increase sample diversity as well as facilitate access to hard-to-reach populations [19] mainly individuals from more rural areas, lacking access to formal elderly community centers [15]. Recommendations from a trusted acquaintance also helped alleviate concerns regarding participation, particularly in a sensitive topic such as EOL preferences.

Sampling continued until data saturation was achieved, with no new themes emerging across the interviews [20].

### Interview tool and procedure

Semi-structured interviews were conducted by the primary researcher, a certified nurse with 20 years’ experience of working with elderly populations in both the Arab and Jewish communities in Israel. The interviews were guided by an interview protocol designed to explore EOL preferences, decision-making processes, and the role of family members. The modular guide, which included both general and targeted questions, was developed in Arabic to align with participants’ linguistic preferences. The interview guide is available as supplement 1.

Interviews were conducted in settings chosen by the participants, including their homes or community centers, to ensure comfort and natural interaction. In order to maintain trust, we were granted permission by the Research Ethics Committee (REC), to forgo signed consent (which in this population may be construed as a forbidding requirement). This has been replaced with oral consent, recorded at the beginning of the interviews’ recording. Each interview lasted between 45 and 90 min, depending on the depth of discussion.

### Data management and translation

Interviews were audio-recorded with participants’ consent and transcribed verbatim by the primary researcher. Transcriptions were translated from Arabic into Hebrew, using a forward-backward translation method to ensure linguistic accuracy and cultural equivalence. The Hebrew version was then translated into English by the second researcher, and this translation was then cross verified with the original Arabic transcription to preserve the authenticity of participants’ narratives.

### Data analysis

Thematic analysis was conducted following Braun and Clarke’s six-phase framework [21], which involved familiarizing with the data, generating codes, searching for themes, reviewing and refining themes, defining and naming them, and producing the final report. This approach enabled systematic identification of patterns within the data, balancing inductive insights with deductive alignment to the study’s objectives.

Initial coding captured key features of the data, which were collated into themes through iterative review. Themes were checked for coherence and refined to ensure they meaningfully addressed the research questions. This process actively involved researcher interpretation, recognizing themes as constructed rather than “emerging” from the data [21]. Two coders were involved in the analysis process in order to enhance credibility and dependability. While the primary researcher coded all transcripts, the second coder focused on verifying a selected subset to validate the consistency of the codes. The analysis integrated both semantic and latent elements, reflecting explicit participant expressions and underlying meanings.

Thematic maps and iterative discussions were used to develop and refine the themes collaboratively [22]. This structured approach provided a comprehensive and credible exploration of participants’ perspectives.

The primary researcher maintained a reflective journal throughout the research process to acknowledge and mitigate potential biases arising from cultural proximity to the participants. Regular consultations with the supervisor further ensured methodological integrity.

### Ethical considerations

This study adhered to rigorous ethical standards to ensure the protection and respect of all participants. Approval was granted by the University of Haifa REC (approval number: 103/22). To accommodate cultural sensitivities and promote participant trust, the Research Ethics Committee (REC) approved a waiver for signed consent. Instead, oral informed consent was obtained and recorded at the beginning of each interview, ensuring voluntary participation while addressing concerns about formal documentation.

## Results

A total of 24 participants (12 elderly individuals and 12 family members) were interviewed, ensuring diversity in religiosity, gender, and socioeconomic status. All members of the elderly cohort lived independently in the community, thus excluding cases of imminently expected EOL decision making.

Participants’ general demographics are presented in Supplement 2.

This paper highlights key findings from our study, focusing on three interwinding themes: perceptions of death, EOL preferences, and barriers to communication. Additional insights, which are beyond the scope of this paper, include themes related to autonomy and truth-telling, trust in the healthcare system and indications of social change.

Main as well as sub-themes discussed in this paper are hereby presented in Table 1.

**Table 1:** Thematic coding table

Theme	Sub-theme	Family Members (*n* = 12)	Age 60 + Group (*n* = 12)	Total (*n* = 24)
Perception of Death	Influence of religion on acceptance of death	10	7	17
	Heaven and Hell	8	10	18
	Death as a natural and inevitable event	6	12	18
	Fear of death	12	3	15
Personal Preferences	Quality of life takes precedence over longevity	12	11	23
	Fear of suffering and being a burden on others	8	6	14
	Desire to die at home surrounded by family	12	12	24
Difficulty Discussing Death	Difficulty talking about death	11	5	16
	Fear of distressing and harming the other party	11	4	15
	Lack of awareness regarding decision-making tools	12	11	23

A table depicting the frequency of each theme’s occurrence among interviewees, categorized by sub-themes and participant groups

### Perceptions of death

The study revealed a complex set of beliefs about death among the elderly Arab participants, shaped by religious teachings and cultural norms. Most elderly participants viewed death as a natural, divinely ordained event to be accepted with dignity. As one participant explained:

“In our faith, death is just the beginning. It is a journey that we must take. I am not afraid because I believe that God has already determined the time for each of us.” (Interviewee 10).

Another participant explained that,

“The Prophet himself showed us how to accept death. He was calm and ready. It reminds us that we should not fear it but embrace it when the time comes.” (Interviewee 1).

For many participants in this study, this world and their current life are perceived as a temporary passage, a transient phase leading to the eternal realm, where they will ultimately meet God—either in Heaven or Hell—based on their deeds and moral conduct. As one of the interviewees articulated

“At the end of days, there will be a day of Judgment, and we will be resurrected. Some will find their fate in heaven, while others will be in hell. “(Interviewee 9).

This deep connection to Islamic narratives about death, which provide comfort and guidance for many elderly individuals, contrasts sharply with the attitudes observed among younger family members. Members of the younger group often expressed fear and discomfort with the topic of death. One younger participant remarked,

“Death is something I try not to think about. It feels like a shadow that we all avoid talking about because it’s so final and terrifying.” (Interviewee 12).

This generational gap suggests that while elderly individuals may find solace in religious teachings, younger family members are less inclined to discuss or engage with the topic due to personal fears [23].

### End-of-Life care preferences

Our study’s elderly participants overwhelmingly voiced a preference for a peaceful death that values quality of life over life extension. The consensus among participants was that they desired to die at home, surrounded by loved ones, rather than in a hospital setting, dependent on medical interventions that might extend their lives without improving their quality.

One elderly participant remarked:

“What’s the point of living longer if each day is filled with pain? I would rather pass away quietly, in my own bed, than in a hospital bed with machines around me” (Interviewee 5).

For many, the idea of spending their final days in a clinical setting was profoundly unappealing. Another participant shared:

“Hospitals are for healing, not for dying. When I die, I want to be in my home, where my family can sit with me, where I feel I belong” (Interviewee 3).

Many elderly participants viewed the preference for quality of life over sheer longevity as aligning with Islamic beliefs regarding suffering and death. One participant encapsulated this belief, stating,

“God has given us life, but He also knows when it should end. If my body is tired, why should I fight what is natural?” (Interviewee 7).

Rather than viewing medical interventions as essential at all costs, these participants saw them as potentially disruptive to a peaceful and dignified death.

Additionally, the desire not to become a burden was a prominent theme in the preferences expressed by elderly participants. Many were acutely aware of the potential emotional and physical toll that their prolonged illness could impose on family members. “I have been strong all my life”, explained one participant. “I don’t want my children to remember me helpless, needing their care. That’s not how I want to leave this world” (Interviewee 9).

This statement reflects a deep-seated concern about preserving family memories and relationships in a positive light, even at life’s end. It also demonstrates a concern for family well-being, where the participants prioritized their loved ones’ emotional health over their own longevity.

The findings further revealed that, for many elderly participants, the ultimate preference for EOL care was not merely a choice between life and death, but rather a preference for how they wished to experience their final days. They expressed a desire for an environment that allows them to preserve dignity and maintain a sense of control, with one participant summarizing it as follows:

“I want to be at home because home is where I’ve lived, where I have memories. I want to die where I feel whole, not in a place that feels cold and unfamiliar” (Interviewee 17).

### Barriers to communication

While the elderly participants articulated preferences for quality of life and “a good death”, many younger family members were unaware of them due to the lack of direct discussions on the topic. In several cases, younger family members assumed that their elders would prefer life-extending treatment options. One younger participant reflected:

“I always thought my father would want to fight to stay with us as long as possible, but I’ve never actually asked him what he would want” (Interviewee 4).

Our study uncovered significant barriers to effective communication between generations. One of the most substantial barriers was cultural taboos surrounding discussions about death. An elderly participant explained this cultural constraint:

“In our community, we don’t talk about death. It’s considered unlucky, like inviting death into our home. So even if I want to discuss it, my children would rather avoid the topic.” (Interviewee 9).

This sentiment was also echoed by younger family members, who felt similarly constrained by cultural expectations. One younger participant stated,

“It feels wrong to bring up death. It’s like I’m telling them to prepare for something that shouldn’t be spoken of until it happens.” (Interviewee 8).

Another significant barrier is the perception that discussing death could cause unnecessary emotional distress for loved ones. Many elderly participants refrained from expressing their wishes out of concern for how their children might react, wishing to shield them from the discomfort and fear associated with contemplating mortality. As one participant remarked:

“I don’t want to burden my children with thoughts about my death. They have their lives, and they don’t need to worry about what I want when my time comes” (Interviewee 13).

This barrier is not solely a product of cultural beliefs but also a reflection of generational differences in how death is perceived and understood. As previously established, while many elderly participants expressed a resigned acceptance of death, viewing it as a natural part of life, younger family members often saw it as something to be feared and avoided. One younger participant explained:

“My parents talk about death as if it’s something peaceful, but for me, it’s terrifying. I can’t imagine them not being here, so I avoid thinking about it altogether.” (Interviewee 12).

This generational gap in perceptions of death complicates efforts to establish open communication, as family members are often hesitant to engage in conversations that might bring these differences to the surface.

Furthermore, the lack of awareness about advance care planning tools, such as durable power of attorney and advance directives, emerged as a significant barrier to fulfilling EOL preferences. Most participants, regardless of age, were unfamiliar with these options and expressed surprise upon learning about them. “I’ve never heard of these things,” one elderly participant said. “If I had known, maybe I would have made some plans.” (Interviewee 3). Many younger family members, who might otherwise encourage advance planning, were similarly unaware of these resources, with one stating:

“I thought that only happens in hospitals, that doctors decide for you. I didn’t know we could decide ahead of time.” (Interviewee 12).

Despite their professional expertise and familiarity with EOL care practices, younger interviewees who worked in healthcare (*n* = 6, for example Interviewee 20) were not immune to the impact of cultural taboos. While these individuals routinely engaged in discussions about advanced directives and EOL decisions in their professional capacities, they never-the-less avoided initiating similar conversations within their own families, reflecting the pervasive influence of cultural norms on personal relationships.

## Discussion

The findings of this study challenge prevailing stereotypes about EOL preferences within Israel’s Arab population, as well as generally shed light on the nuanced interplay between cultural, religious, and individual factors effecting EOL decision making in the Muslim population. It is often assumed– both by the younger family members we interviewed and health providers around the world [24–26]– that Arab patients prioritize life extension due to cultural and religious imperatives. Yet our study reveals a more complex reality, where many of our elderly study’s participants expressed preferences for a peaceful death, prioritizing dignity and family presence over sheer longevity.

### Cultural and religious dimensions

Islamic teachings provide a significant framework for understanding participants’ attitudes toward EOL care. In Islamic tradition, preserving life is paramount, yet there is also an acceptance of death when medical intervention merely prolongs suffering [11]. Many Muslim scholars argue that in such cases, allowing the natural process of dying to take its course may not only be permissible but also a compassionate approach [14, 15]. This perspective is not merely justified but strongly encouraged, as Islamic tradition emphasizes providing comfort and pain relief to terminally ill individuals in a peaceful environment, surrounded by family and friends [15].

This nuanced understanding is reflected in the EOL preferences of our study participants, which aligns with studies in some Muslim-majority countries, where patients and families prioritize relief from suffering over life extension, particularly in terminal illness cases [27, 28], Similarly, recent studies indicate that EOL preferences among Muslim minority communities often emphasize quality of life, comfort, and adherence to religious beliefs that uphold dignity and the presence of family during one’s final days [29, 30].

### Communication barriers and generational differences

In Western societies, autonomy and self-determination often dominate EOL decision-making [31]. In contrast, collectivist societies, including many in the Middle East and Asia, emphasize family involvement and shared decision-making [32, 33]. Within Israel’s Arab population, this collectivist perspective is evident, as family members play a central role in navigating EOL decisions [9].

Findings published by Bodas [34] further illustrate this trend, showing that the proportion of individuals prioritizing personal autonomy in EOL decisions is lower among the Arab population in Israel (19%) compared to Israeli Jews (33%). This difference underscores the stronger inclination within the Arab community toward collective decision-making, where individuals are more likely to involve family members rather than make choices independently. Participants in our study also emphasized the dual importance of familial involvement and adherence to religious values, highlighting the interconnectedness of personal, familial, and spiritual dimensions in EOL care.

In collectivist societies, where EOL decisions are made within the family unit, effective communication among family members is particularly crucial. Yet, our study uncovered a significant communication gap between elderly individuals and their families regarding EOL preferences.

The avoidance of EOL discussions is not unique to Israel’s Arab community. Research in other traditional societies also highlights cultural barriers to discussing death and dying [25]. For example, studies in Palestinian families reveal that patients often desire autonomy in EOL decisions but refrain from expressing their preferences due to social constraints [35]. Cultural taboos surrounding discussions about death often prevent open dialogue, leaving family members unaware of their elders’ true desires [23, 26, 36].

Additionally, as both our research findings and existing literature indicate, the fear of becoming a burden on family and loved ones is a significant theme in the experiences of patients and their families in the context of EOL care [30]. More than half of the interviewees from both groups expressed concerns about the impact their care needs might have on their families, recognizing the potential emotional and practical consequences. Since articulating such concerns directly to those who would bear the burden can be particularly challenging, these conversations are often avoided, further limiting open discussions about EOL preferences [30].

Notably, our research uncovered a unique basis further exacerbation the lack of dialog: While elderly participants viewed death as a natural transition, consistent with Islamic teachings, welcoming death when their time comes as they will be reunited with God in Heaven, younger family members often perceived death with fear and avoidance. This generational divide further hinders effective communication and contributes to decision-making based on assumptions rather than clarity.

These findings underscore the need to address cultural taboos and generational differences to facilitate open dialogue within families.

### Advance care planning and policy implications

The lack of awareness about advance care planning tools, such as durable power of attorney and advanced directives, emerged as a significant barrier in this study. Most participants, regardless of age, were unfamiliar with these tools, reflecting broader trends in minority communities worldwide. Studies indicate that cultural minorities often complete advance directives at lower rates than majority populations, viewing them as intrusive to family dynamics [32, 37]. For Israel’s Arab population, promoting awareness of advance care planning within a culturally sensitive framework is essential to bridging this gap.

Islamic perspectives on advance directives provide a valuable entry point for such efforts. Islamic teachings recognize the validity of advance directives, and recent years have witnessed a robust discourse on their role and ethical considerations [38]. While some Muslim scholars argue that advance directives align with religious teachings [15], others express concerns about their implications for patient care [9]. Policymakers and healthcare providers must navigate these differing views to ensure that advance care planning respects both individual autonomy and cultural values. Successful examples from Muslim-majority countries, such as Malaysia, demonstrate how culturally tailored approaches can enhance acceptance of advance directives while preserving family harmony [39].

### Bridging assumptions and preferences

In a multicultural society like Israel, the coexistence of diverse value systems can create ethical challenges, particularly in healthcare. Cultural and religious differences between patients, families, and physicians often lead to disparities in decision-making, underscoring the need for a more inclusive approach to biomedical ethics [40].

The findings of this study highlight the critical need for healthcare providers worldwide to challenge assumptions about minority populations’ EOL preferences. Training in culturally sensitive communication is essential to fostering trust and facilitating open discussions between patients, families, and healthcare professionals. By addressing stereotypes and promoting patient-centered care, healthcare providers can align EOL decisions with individual preferences rather than defaulting to generalized cultural assumptions.

Moreover, while the Israeli healthcare system does not sufficiently facilitate advance EOL planning, across all cultural groups [29], addressing this shortcoming is particularly crucial in the context of the Arab population. Integrating family involvement into EOL care policies can help balance collectivist cultural values with individual autonomy. To achieve this, discussions about EOL preferences should take on a more structured and intentional manner [32]. Encouraging families to engage in advance care planning discussions—while providing them with the necessary tools and resources—can empower them to make informed, compassionate decisions. These efforts must be supported by public health campaigns that destigmatize death-related conversations and promote awareness of available options for EOL care.

### Strengths and limitations

This study addresses a gap in current knowledge by exploring EOL care preferences in the Arab population in Israel, an under-researched group. The inclusion of participants from diverse sub-regions enhances the cultural and demographic relevance of the findings. By incorporating both elderly individuals and their family members, the study provides a comprehensive view of elderly individuals’ and their family caregivers’ perspectives on EOL care. The rigorous application of thematic analysis ensures methodological transparency, and the culturally sensitive design, including interviews conducted in Arabic, fosters trust and authenticity in participants’ narratives.

Yet several potential biases should be acknowledged, mainly translation, cultural interpretation and interviewer influence biases. The forward-backward translation process, coupled with bilingual cross-verification, was employed to preserve cultural and linguistic accuracy. Despite these measures, some cultural nuances may have been challenging to fully capture in translation. Additionally, the interviewer’s cultural proximity to participants facilitated rapport but may have unintentionally influenced responses. Reflexivity practices were used to mitigate these effects. Finally, recall bias, though of lesser importance in an attitudes-centered interview, was addressed by focusing on recurring patterns in participants’ narratives, ensuring that findings reflect consistent themes.

In addition, while participants were sampled from three distinct sub-regions to capture diversity, the use of purposive and snowball sampling may limit generalizability, both for underrepresented religious minorities in Israel, such as Arab Christians or Druze, and as a comprehensive case study for Muslim minorities worldwide. Additionally, the focus on family dynamics provided valuable insights but may have overshadowed the perspectives of individuals with limited family involvement or a preference for autonomous decision-making. Finally, the analysis emphasized the gap between assumptions and actual preferences in EOL care. Additional themes, such as broader cultural shifts or systemic influences, emerged during data analysis, yet were not included in this manuscript due to scop limitations.

## Conclusions

This study highlights the complex and nuanced nature of EOL care preferences within cultural and religious minority populations, focusing on Israel’s Arab– predominantly Muslim– community. Contrary to widespread assumptions, many of the study’s elderly participants prioritized quality of life and dying at home with family members present over life-extending medical interventions. These preferences challenge the stereotype that minority populations universally favor aggressive treatments, typically administered in hospital settings, due to cultural or religious beliefs.

Cultural and religious minorities face unique barriers to achieving patient-centered EOL care. These barriers include a lack of awareness of advance care planning tools and cultural taboos surrounding discussions about mortality. While this study revealed generational divides in attitudes toward death among the Arab population in Israel, further research is needed to explore whether similar patterns exist in diverse global contexts.

The findings underscore the need for targeted policy interventions, such as public health campaigns to promote advance care planning within minority communities. These efforts should incorporate culturally relevant educational materials and encourage open family dialogues about EOL preferences. Additionally, healthcare providers must receive training in culturally competent communication to foster trust and facilitate meaningful discussions with patients and their families.

Lessons from this study resonate with other collectivist societies where family involvement is central to decision-making. Policymakers and healthcare systems must adopt flexible frameworks that integrate diverse cultural values without compromising individual autonomy.

By bridging the gap between assumptions and actual preferences, healthcare systems can ensure that EOL care is both compassionate and respectful of cultural diversity. These efforts will not only improve the quality of care for minority populations but may also promote equitable healthcare practices on a global scale.

## Electronic supplementary material

Below is the link to the electronic supplementary material.

Supplementary Material 1

Supplementary Material 2

## Data Availability

The datasets used and/or analysed during the current study are available from the corresponding author on reasonable request.

## References

[1] Schweda M, Schicktanz S, Raz A, Silvers A. Beyond cultural stereotyping: views on end-of-life decision making among religious and secular persons in the USA, Germany, and Israel. BMC Med Ethics. 2017;18(1):1–11. 28212642 10.1186/s12910-017-0170-4 PMC5316158

[2] The Dying patient Law. 2005. Unofficial translation into English available at https://www.medethics.org.il/article/the-dying-patient-law-2005-2/

[3] The Legal Capacity. and Guardianship Law, 2016.

[4] The Patient’s Rights Law. 1996. Unofficial translation into English available at https://hamoked.org/files/2013/155880_eng.pdf

[5] Steinberg A, Sprung CL, Steinberg A. End of life: National legislations: the dying patient: new Israeli legislation. Intensive Care Med. 2006;32:1234–7. 16718456 10.1007/s00134-006-0186-6

[6] Inthorn J, Schicktanz S, Rimon-Zarfaty N, Raz A. What the patient wants… Lay attitudes towards end-of-life decisions in Germany and Israel. Med Health Care Philos [Internet].2015; 18(3):329–40. Available from: 10.1007/s11019-014-9606-5. 10.1007/s11019-014-9606-5 25344758

[7] Arab Society in Israel. Yearbook 2023– The Israel Democracy Institute (Heb)available at https://www.idi.org.il/books/53397

[8] Israeli central bureau of statistics: Social survey 2021 [Internet]. 2023 Mar. Available from: www.cbs.gov.il

[9] Brodsky G, Azaiza F. Changes in the Arab family and development of services for elderly Arabs in Israel over the last decade. Gerontology. 1995;(70):69–80. (Heb).

[10] Bloomer MJ, Al-Mutair A. Ensuring cultural sensitivity for Muslim patients in the Australian ICU: Considerations for care. Australian Critical Care [Internet]. 2013;26(4):193–6. Available from: 10.1016/j.aucc.2013.04.003 10.1016/j.aucc.2013.04.003 23693083

[11] Fang ML, Sixsmith J, Sinclair S, Horst G. A knowledge synthesis of culturally- and spiritually-sensitive end-of-life care: Findings from a scoping review. BMC Geriatr [Internet]. 2016;16(1):1–14. Available from: 10.1186/s12877-016-0282-6 10.1186/s12877-016-0282-6 PMC4872365 27193395

[12] Chamsi-Pasha H, Albar MA. Ethical dilemmas at the end of life: Islamic perspective. J Relig Health. 2017;56:400–10. 26797682 10.1007/s10943-016-0181-3

[13] Miklancie MA. Caring for patients of diverse religious traditions: Islam, a way of life for Muslims. Home Healthc Nurse. 2007;25(6):413–7. 17556925 10.1097/01.NHH.0000277692.11916.f3

[14] Bülow HH, Sprung CL, Baras M, Carmel S, Svantesson M, Benbenishty J, et al. Are religion and religiosity important to end-of-life decisions and patient autonomy in The ICU? The ethicatt study. Intensive Care Med. 2012;38(7):1126–33. 22527070 10.1007/s00134-012-2554-8

[15] Braun K, Pietsch J, Blanchette P. Cultural Issues in End-of-Life Decision Making [Internet]. Thousand Oaks, California: SAGE Publications, Inc.; 2000. Available from: https://sk.sagepub.com/book/edvol/cultural-issues-in-end-of-life-decision-making/toc

[16] Smith JA, Osborn M. Interpretative phenomenological analysis. In: Smith JA, editor. Qualitative psychology: a practical guide to research methods. London: Sage; 2008. pp. 53–80.

[17] Patton M. Qualitative research evaluation methods. 3 Rd. Sage Publications. Sage; 2002.

[18] Biernacki P, Waldorf D. Snowball Sampling: Problems and Techniques of Chain Referral Sampling. Sociol Methods Res [Internet]. 1981;10(2):141–63. Available from: 10.1177/004912418101000205

[19] Parker C, Scott S, Geddes A. Snowball Sampling [Internet]. London: SAGE Publications Ltd; 2019. Available from: https://methods.sagepub.com/foundations/snowball-sampling

[20] Fusch PI, Ness LR. Are we there yet? Data saturation in qualitative research. Qual Rep. 2015; 20 (9): 1408–16. 2015.

[21] Braun V, Clarke V. Using thematic analysis in psychology. Qual Res Psychol. 2006;3(2):77–101.

[22] Kiger ME, Varpio L. Thematic analysis of qualitative data: AMEE guide 131. Med Teach. 2020;42(8):846–54. 32356468 10.1080/0142159X.2020.1755030

[23] Karim K. Informing cancer patients: truth telling and culture. Cancer Nurs Pract. 2003;2(3):23–31.

[24] Sprung CL, Maia P, Bulow HH, Ricou B, Armaganidis A, Baras M et al. The importance of religious affiliation and culture on end-of-life decisions in European intensive care units. Intensive Care Med [Internet]. 2007 [cited 2022 Jan 1];33(10):1732–9. Available from: https://pubmed.ncbi.nlm.nih.gov/17541550/10.1007/s00134-007-0693-0 17541550

[25] Tohmé A, Younés H, Mouhawej MC. An overview on palliative care and the end of life in Lebanon. Results of a survey among university students. Journal Medical Libanais [Internet]. 2016 [cited 2022 Jan 1]; 66. Available from: https://www.researchgate.net/publication/324809441_An_overview_on_palliative_care_and_the_end_of_life_in_Lebanon_Results_of_a_survey_among_university_students

[26] Mouhawej MC, Maalouf-Haddad N, Tohmé A. Cultural challenges in implementing palliative services in Lebanon. palliative medicine and hospice care -. Open J. 2017;SE(1):S15–8.

[27] Goldwin A. The ethics of assisting people who wish to die. Gerontol Geriatr. 2014;4:45–64. (Heb).

[28] Leong M, Olnick S, Akmal T, Copenhaver A, Razzak R. How Islam Influences End-of-Life Care: Education for Palliative Care Clinicians. J Pain Symptom Manage [Internet]. 2016;52(6): 771–774.e3. Available from: 10.1016/j.jpainsymman.2016.05.034 10.1016/j.jpainsymman.2016.05.034 27810572

[29] Karako-Eyal N, Gilbar R, Peled-Raz M. The dying patient act 2005 ten years on: law, ethics & medical practice. Tel Aviv Law Rev. 2018;41:73. (Heb).

[30] Abdullah R, Guo P, Harding R. Preferences and experiences of Muslim patients and their families in Muslim-Majority countries for End-of-Life care: A systematic review and thematic analysis. Journal of Pain and Symptom Management. Volume 60. Elsevier Inc.; 2020. pp. 1223–e12384. 10.1016/j.jpainsymman.2020.06.032 32659320

[31] Malek MM, Abdul Rahman NN, Hasan MS, Haji Abdullah L. Islamic considerations on the application of patient’s autonomy in End-of-Life decision. J Relig Health. 2018;57:1524–37. 29417395 10.1007/s10943-018-0575-5

[32] Johnstone Mjane. Ethics and advance care planning in a culturally diverse society. J Transcult Nurs. 2009;20(4):405–16. 19597187 10.1177/1043659609340803

[33] Zeldes N. Three thirsts of new Christians (Neophytes) from Sicily in the age of expulsion: between Jewish past and Christian present. Pa’amim: Q Study Jew Communities East. 2005;103:101–28. (Heb).

[34] Bodas M, Velan B, Kaplan G, Ziv A, Rubin C, Peleg K. Assisted life termination and truth telling to terminally ill patients– a cross-sectional study of public opinions in Israel. Isr J Health Policy Res. 2020;9(1). 10.1186/s13584-020-00419-9 PMC7586668 33106184

[35] Gamliel T. The macabre style: coping with death in old age. Megamwt. 2003;42(2):240–62.

[36] Alrimawi I, Saifan AR, Abdelkader R, Batiha AM. Palestinian community perceptions of do-not-resuscitation order for terminally ill patients: A qualitative study. J Clin Nurs. 2018;27(13–14):2719–28. 28557015 10.1111/jocn.13905

[37] Bito S, Matsumura S, Singer MK, Meredith LS, Fukuhara S, Wenger NS. Acculturation and end-of-life decision making: comparison of Japanese and Japanese-American focus groups. Bioethics. 2007;21(5):251–62. 17845470 10.1111/j.1467-8519.2007.00551.x

[38] Al-Jahdali H, Baharoon S, Al Sayyari A, Al-Ahmad G. Advance medical directives: a proposed new approach and terminology from an Islamic perspective. Med Health Care Philos. 2013;16(2):163–9. 24571002 10.1007/s11019-012-9382-z

[39] Malek MM, Saifuddeen SM, Abdul Rahman NN, Yusof ANM, Abdul Majid WR. Honouring wishes of patients: an Islamic view on the implementation of the advance medical directive in Malaysia. Malaysian J Med Sci. 2021;28(2):28–38. 10.21315/mjms2021.28.2.3 PMC8075601 33958958

[40] İlkiliç İ. Bioethical issues in the relationship between Muslim patient and non-Muslim physician. Biomed Ethics. 2000;5:125–30. 12755117

