# Supplementary material for: End of life care preferences in the Arab population in Israel– bridging the gap between unfounded assumptions and autonomous wishes
Source: BMC Med Ethics. 2025 Apr 4;26:42. doi: 10.1186/s12910-025-01201-9 (PMC11969688; doi:10.1186/s12910-025-01201-9)
Supplement: Supplementary file 1 — Supplementary Material 1 [file 12910_2025_1201_MOESM1_ESM.docx]

**Supplement 1**

**Interview Guide with Elderly Patients**

**Warm-Up and Introduction**

Hello, and thank you for agreeing to meet with me.

My name is Murad Said Ahmad, a master's student in Health Systems Management at the University of Haifa.

We will be discussing the topic of death and end-of-life issues within the Arab population in Israel. We are recording the interview to accurately document your views and opinions. It’s important to emphasize that there are no right or wrong answers—what matters is your perspective on the subject.

1. Please tell me about yourself: Who are you? How old are you, and what do you do in life?
2. What are some significant things you have done in life that you would like to share?

**Opening Questions on Health and Death**

1. How would you describe your health condition?

**Discussion on End-of-Life in the Arab Community**

1. Can you please share if there is a conversation about death in the Arab community?
2. To what extent do you think end-of-life and death concerns are part of the discourse among people of your age?
3. Some say that people do not talk about death. What is your opinion on this, and why does it happen?
4. Would you like people to talk about it more openly and transparently? How would you prefer this to be done?
5. If, God forbid, there were bad diagnoses related to you, how would you want to be informed, if at all? Would you want people to discuss it with you?

**Perceptions About Decision-Making at End-of-Life**

1. From your worldview, what do you prioritize more—quality of life or longevity? Please explain why you see it that way.
2. If a doctor told you there was no life-saving treatment and only supportive care was available, what would you say?
3. What does "end of life" mean to you? How do you think others perceive the concept of end-of-life?
4. What is death to you, and how do you interpret and understand it?
5. What do you think influences perceptions of life and death—life experiences, religious beliefs, or other factors?
6. Where do you envision this happening, and where would you prefer it not to occur?

**Family Involvement in Decision-Making**

1. Please tell me about your relationship with your family. What do you usually do with them, how often do you meet, and do you feel free to discuss sensitive topics with them? I’d love for you to provide examples.
2. Who in your family are you closest to, and why do you consider this person the closest?
3. Some say the amount and quality of conversations with family decreases over time. What is your view on this?
4. Can you share a conversation you had about end-of-life or death with your family members?

**Decision-Making Practices in the Arab Society**

1. How are decisions typically made in Arab society?
2. Some believe that as people age, decisions are made differently than when they were younger. What is your opinion on this?

**Decision-Makers at End-of-Life**

1. Have you shared your end-of-life preferences with those around you?
2. Who do you think should make end-of-life decisions, and why?
3. Who do you think actually makes the end-of-life decisions, and how are these decisions made?
4. Who would you prefer to be involved in end-of-life decision-making, and in what way?
5. Who do you believe will actually be involved, and how will it look (dominant, consultative)?

**Awareness of Advance Planning and Giving Advance Directives**

1. Have you heard of advance directives and powers of attorney? Is there any discussion about it? What are your thoughts?
2. What instructions, if any, would you give, and why?
3. Would you appoint someone, and if so, who and why?

**Interview Guide with Family Members**

**Warm-Up and Introduction**

Hello, and thank you for agreeing to meet with me.

My name is Murad Said Ahmad, a master's student in Health Systems Management at the University of Haifa.

You are likely someone who will play a central role in end-of-life decision-making for your relative, and we will discuss death and end-of-life topics in the Arab population in Israel. We are recording the interview to accurately document your views and opinions. It’s important to note that there are no right or wrong answers—what matters is your perspective on the subject.

(Relative = Father / Mother / Other, as appropriate)

1. Please tell me about yourself: Who are you? How old are you, and what do you do in life?
2. What significant things have you done in life that you would like to share?

**Opening Questions on Health and Death**

1. How would you describe your relative’s health condition?

**Discussion on End-of-Life in the Arab Community**

1. Can you please share if there is a conversation about death in the Arab community?
2. To what extent do you think end-of-life and death issues are part of the discourse among your relative’s age group?
3. Some say that people do not talk about death. What is your opinion on this, and why does it happen?
4. Would you like people to talk about it more openly and transparently? How would you prefer this to be done?
5. If, God forbid, there were bad diagnoses related to your relative, would you want them to be informed, and do you know their preferences on this matter?

**Perceptions About Decision-Making at End-of-Life**

1. In your view, what is more important to your relative—quality of life or longevity? Please explain why you think so.
2. If a doctor told your relative there was no life-saving treatment and only supportive care was available, how do you think your relative would respond?
3. What does "end of life" mean to you? How do you think others view the concept?
4. What is death to you, and how do you interpret and understand it?
5. What do you think influences perceptions of life and death?
6. Where does your relative envision this happening, and where would they prefer it not to occur?

**Family Involvement in Decision-Making**

1. Please tell me about your relationship with your relative. What do you usually do together, how often do you meet, and do you feel free to discuss sensitive topics with them? Please give examples.
2. Who do you think is closest to your relative, and why do you think so?
3. Some say the amount and quality of conversations with family decrease over time. What is your view on this?
4. Can you share a conversation you had with your relative about end-of-life or death?

**Decision-Making Practices in the Arab Society**

1. How are decisions typically made in Arab society and within your family?
2. Some believe that as people age, decisions are made differently than when they were younger. What is your opinion on this?

**Decision-Makers at End-of-Life**

1. Has your relative shared their end-of-life preferences with you?
2. Who do you think should make end-of-life decisions for your relative, and why?
3. Who do you think makes the decisions, and how are these decisions made?
4. Who do you think your relative prefers to be involved in end-of-life decision-making, and in what way?
5. Who do you think your relative believes will be involved, and how will it look (dominant, consultative)?

**Awareness of Advance Planning and Giving Advance Directives**

1. Have you heard of advance directives and powers of attorney? Is there any discussion about it? What are your thoughts?
2. What instructions do you think your relative would give, if any, and why?
3. Do you think your relative would appoint someone? If so, who and why?
