# Supplementary material for: End of life care preferences in the Arab population in Israel– bridging the gap between unfounded assumptions and autonomous wishes
Source: BMC Med Ethics. 2025 Apr 4;26:42. doi: 10.1186/s12910-025-01201-9 (PMC11969688; doi:10.1186/s12910-025-01201-9)
Supplement: Supplementary file 2 — Supplementary Material 2 [file 12910_2025_1201_MOESM2_ESM.docx]

**Supplement 2 – Participants' Demographics**

**Table S1: Characteristics of Participants in Group 1 – Elderly**

| **Interview #** | **Corresponding to participant #** | **Gender** | **Age range** | **Marital Status**  (D = Divorcee,  M = Married,  W = Widowed) | **Number of Children** |
| --- | --- | --- | --- | --- | --- |
| 1 | 2 | Male | 76-80 | M | 6-10 |
| 3 | 4 | Male | 61-65 | M | 6-10 |
| 5 | 6 | Female | 76-80 | W | 0-5 |
| 7 | 8 | Male | 86-90 | M | 11-15 |
| 9 | 10 | Female | 66-70 | D | 6-10 |
| 11 | 12 | Male | 91-95 | M | >15 |
| 13 | 14 | Male | 76-80 | M | >15 |
| 15 | 16 | Female | 81-85 | W | 6-10 |
| 17 | 18 | Female | 61-65 | M | 0-5 |
| 19 | 20 | Male | 71-75 | M | 6-10 |
| 21 | 22 | Male | 61-65 | M | 0-5 |
| 23 | 24 | Male | 86-90 | W | 6-10 |

**Table S2: Characteristics of Participants in Group 2 – Family Members**

| **Interview #** | **Corresponding to participant #** | **Gender** | **Age** | **Marital Status**  (M = Married,  S = Single) | **Number of Children** | **Relationship to Elderly Participant** |
| --- | --- | --- | --- | --- | --- | --- |
| 2 | 1 | Female | 51-55 | M | >3 | Daughter |
| 4 | 3 | Female | 31-35 | M | >3 | Daughter |
| 6 | 5 | Female | 51-55 | M | 2-3 | Sister |
| 8 | 7 | Male | 41-45 | M | 2-3 | Son |
| 10 | 9 | Male | 46-50 | M | >3 | Son |
| 12 | 11 | Male | 46-50 | M | 2-3 | Son |
| 14 | 13 | Female | 61-65 | M | >3 | wife |
| 16 | 15 | Male | 26-30 | S | 0-1 | Son |
| 18 | 17 | Male | 36-40 | M | 2-3 | Son |
| 20 | 19 | Female | 31-35 | M | 2-3 | Daughter |
| 22 | 21 | Female | 26-30 | S | 0-1 | Daughter |
| 24 | 23 | Male | 26-30 | S | 0-1 | Son |
